# Editorial: Telehealth and connected health: equity and access to care

**DOI:** 10.3389/fdgth.2024.1399325

**Published:** 2024-04-04

**Authors:** Mirna Becevic, Ateev Mehrotra

**Affiliations:** ^1^ Department of Dermatology, School of Medicine, Missouri Telehealth Network, University of Missouri, Columbia, SC, United States; ^2^ Department of Health Care Policy, Harvard Medical School, Harvard University, Cambridge, MA, United States

**Keywords:** telemedicine, telehealth, project ECHO, equity and access, mHealth

**Editorial on the Research Topic** Telehealth and connected health: equity and access to care

Rural patients frequently cannot access the healthcare they need. An array of factors underly these access barriers including workforce shortages, reimbursement issues, and transportation constraints. The resulting disparities between rural and urban patients are well documented in the literature (1–4). Other vulnerable populations, such as racial and ethnic minorities, under- or un- insured, patients with certain medical conditions, and socioeconomically disadvantaged, also face similar barriers in access to healthcare (5).

Ensuring that all patients have equitable access to high-quality care poses a significant challenge. In recent years, healthcare transformations due to the rapid advancement of mobile health technologies revolutionized the delivery and accessibility of medical services and reshaped traditional models of care. The COVID-19 pandemic highlighted the utility of disruptive innovation technologies such as telehealth in helping patients access care while minimizing unnecessary exposure to the virus.

In this research topic, Telehealth and Connected Health: Equity and Access to Care, our goal was to highlight studies covering a variety of telehealth applications, including direct patient care, virtual continuing education, and digital health data collection. These studies can help inform initiatives with workforce support and development, healthcare policy, and patient experience. The final Research Topic includes 4 articles: 2 original research articles, 1 community case study, and 1 brief research report.

Findings from Mateus et al. on the utility of a pediatric emergency telemedicine network highlight the importance of a visual component in a telemedicine visit. Telemedicine improved efficiency of communication between healthcare providers and patients, while enabling tertiary hospitals to connect to a trusted, specialized healthcare center reflecting a pre-established sense of trust. When utilized in this way, telemedicine enhances established healthcare delivery mechanisms and improves patient and caregiver experience. In fact, Mateus et al. reported that patients viewed telemedicine only as a means of delivery, not shifting the focus from the patient care. Telemedicine program was well accepted by patients and physicians for the treatment of critically ill pediatric patients in emergency departments, with immediate benefits of rapid connection and improved communication. Authors indicated operational challenges, such as unfamiliarity with the program, lack of buy-in, and perceptions about the time constraints that may need to be address systematically to further expand telemedicine applications.

In their review of published literature on the applications of digital health (telemedicine, remote monitoring, text messaging, wearable electronic devices, etc.) in the United Kingdon (UK), Geifman et al. found older and middle-aged adults use digital health applications primarily for addressing cardio-metabolic and psychiatric conditions, while the most prevalent disease focus for children and young adults is asthma.

Black et al.’s study on the thematic analysis of dermatology ECHO (Extension for Community Healthcare Outcomes) participant surveys can help inform best practices for the development of continuing education didactics interventions within the tele-dermatology ECHO network. Authors identified three major themes—Likes, Dislikes, and Motivations for participating. Participants valued the program's structure (didactic presentation followed by case based learning), as well as the ability to improve dermoscopy skills and confidence in diagnosing skin cancer (Likes). The need for additional content was listed as a common Dislike by the participants. Participants shared their desire for improvement and to gain proficiency in dermoscopy as well as an overall interest in dermatology (Motivation for participating).

Adults, aged ≥60 years, who used chatbots (virtual conversational agents) for health data submission reported perceived ease of use and satisfaction, as reported by Wilczewski et al. Participants reported the use of chatbots was similar to talking with a human, however, they indicated preferences to in-person form completion when additional questions arose. Some older adults also reported concerns over privacy violations and lack of trust when using the chatbot.

The insights gained from these studies highlight the impact of connected health and telehealth on access to care and health equity (6). Findings presented in this Research Topic collectively emphasize the multifaceted implications of virtual technologies in shaping healthcare industry. Telemedicine plays pivotal role in fostering efficient communication between clinicians and patients by providing necessary visual components. Seamless integration and operations support, however, are necessary to streamline care delivery and enhance patient, caregiver and clinician experience and satisfaction.
